# Supplementary material for: Management of postnatal depression: A systematic review of clinical practice guidelines
Source: Glob Ment Health (Camb). 2025 Oct 16;12:e122. doi: 10.1017/gmh.2025.10075 (PMC12641302; doi:10.1017/gmh.2025.10075)
Supplement: Durrani et al. supplementary material [file S2054425125100757sup001.zip › SR_Protocol_21.03.2024_AD.docx]

Systematic Review Protocol

| Title of the review | Management of Postnatal Depression: A Systematic Review of Clinical Practice Guidelines |
| --- | --- |
| **First reviewer** | Aliya Durrani. PhD Scholar, School of Medicine, Keele University, UK |
| **Other reviewers (with role/contribution in the review)** | Prof. Saeed Farooq. Lead Supervisor, School of Medicine, Keele University, UK  Dr Tom Kingstone. Co-Supervisor, School of Medicine, Keele University, UK  Dr Ram Bajpai. Co-Supervisor, School of Medicine, Keele University, UK  Dr Zohaib Khan. Co-Supervisor, Khyber Medical University, Pakistan  Dr Mifrah Rauf Sethi, Peshawar Medical College, Pakistan |
| **Clinical Portfolio Group** |  |
| **Funding source** | This review is a part of Doctoral Studies under the NIHR-funded project titled “Cognitive Therapy of Depression Treatment in TB Patients (The CONTROL). CONTROL NIHR201773/ PID-200016 |
| **PROSPERO registration number** |  |

| **Amendments to the protocol** |  |
| --- | --- |

| 1. **Background to review**  Brief introduction to the subject of the review, including rationale for undertaking the review and overall aim |
| --- |
| Postnatal depression (PND) is one of the most prominent illnesses during the postpartum period when the mother is physically healing after giving birth. The Diagnostic and Statistical Manual of Mental Disorders, 5th Edition (DSM-5), states that “Postpartum Depression” (a class of major depressive disorders) is characterized by symptoms appearing 4–6 weeks after delivery and peak 2-3 months after childbirth (Hobeika et al., 2023). The symptoms include disturbances in appetite and sleep, energy loss, the sensation of guilt, diminished attentiveness, and plausible suicidal thoughts. PND is a well-known pregnancy complication and the second most common cause of disability, after HIV/AIDS. An estimated 10-15% of women worldwide are thought to be affected by PND (Cheng et al., 2023). Recent data suggest that PND is more prevalent in low-middle-income countries (LMIC), where it occurs at a prevalence of 7-33%, compared to high-income nations, where it is 13-19% (Dadi et al., 2020).PND is responsible for an increase in healthcare costs, compromises the quality of life, and can have detrimental repercussions on a child's development, including behavioural disturbances, cognitive performance deficits, and attachment insecurities in the early years. It has also been linked to higher rates of partner depression and marital conflict, which may impact children's developmental outcomes. PND has several risk factors, including a history of psychiatric disorders in the patient, psycho-social issues like marital problems and a decline in social support, the baby's sex, socioeconomic issues, and medical problems like complications during delivery and pregnancy and poor sleep (Dadi et al., 2020, Ahmadpour et al., 2023).  The key barriers to treatment implementation in LMIC are stigma, inadequate budget allocation, weakened health systems, and a lack of guidelines for managing PND in resource-constrained settings. Effective clinical practice guidelines (CPGs) are essential to overcome these challenges (Place et al., 2016). CPGs provide a summary of the most recent evidence in a particular area of clinical practice. They are supported by professional organizations as well as the government due to have the potential to reduce the variability in practice. The Institute of Medicine (US) defines CPGs as ‘statements that include recommendations developed or endorsed by authoritative medical or health organizations to assist practitioners with decisions intended to optimize patient care’ (Keating et al., 2017). CPGs are systematically developed, based on evidence from a rigorous systematic review, and an assessment of the benefits and harms of alternative care options (Keating et al., 2017). However, these systematic reviews use varied inclusion criteria, quality requirements, origin countries, search methods, and publication dates. To raise the standard of care given to the patients, a summary of the recommendations made by these CPGs on PND is required (Hui et al., 2019). In addition to their usefulness in clinical settings, CPGs may also have an impact on policy to ensure consistency and uniformity of care delivered in various healthcare settings. Literature suggests that evidence-based guidelines have the potential to improve the care of the mother and newborn and minimize their short- and long-term morbidity.  The CPGs provide a gold standard for the management of postnatal depression in different healthcare settings. It can bring an improvement in the quality of services provided and the safety of medication use in this period (Abdoli Najmi et al., 2023). However, it appears that these are not available in most LMICs due to a lack of resources and skills. Therefore, the healthcare providers  (HCPs) in these countries depend on CPGs developed by international organizations and professional bodies in higher-income countries (HICs) (Olayemi et al., 2017). However, CPGs established in HICs are difficult to use in the LMIC setting due to major differences in socioeconomics, demographics, culture, and pharmacodynamics. The healthcare requirements of LMICs, the resources available to healthcare professionals there, and the challenges they encounter are likewise unknown to these experts. Additionally, CPGs from HICs are resource-neutral and are probably unusable in LMICs because of insufficient resources and a lack of relevance. All these factors result in conflicts in recommendations intended for use in LMICs.  There is evidence that local guidelines are more likely to be effective due to the sensitive nature of postnatal depression. So, the best course of action is to involve the local stakeholders and experts to adapt the currently available guidelines for PND that will suit the needs and cultural practices of the local community and will also be a cost-effective means of achieving the highest standard of care (Johnston et al., 2019, Olayemi et al., 2017). According to the Guidelines International Network (G-I-N), guideline adaptation is defined as “the systematic approach to the modification of a guideline(s) produced in one cultural and organizational setting for application in a different context”. This method serves as a key vehicle for the local decision-makers to produce contextually relevant and robust guidance for their setting. This process of adaptation considers the local evidence such as specific health questions, priorities, legislation, policies, and most importantly resources, which gives a sense of ownership to the local users which can bring a significant improvement in the acceptance and adherence to the adapted recommendation. Adaptation of evidence-based guidelines from HICs for the LMICs will be a significant step in advancing the healthcare systems in these resource-constrained countries (Wang et al., 2018). Therefore, it is of utmost importance to systematically review the available CPGs with a focus on both pharmacological and non-pharmacological interventions. Because there are also variations in the purpose, content, and complexity of the presentation of recommendations provided by them. We aim to review the CPGs available for the prevention and management of PND, collate the guidelines and recommendations, and consider the feasibility of adapting them for LMIC settings. The findings from this review will play a pivotal role in comparing the nature and scope of these CPGs in the context of LMICs. |

| **2.** **Specific objectives/questions the review will address** |
| --- |
| Research Question:   - What are the current clinical practice guidelines available for managing Postnatal depression (PND)? - What are the specific recommendations reported by these guidelines for the management of PND and the quality of evidence underlying these recommendations? - What are the pharmacological and non-pharmacological interventions recommended for the treatment of PND?   Research Objective:   - To identify the existing clinical practice guidelines for the management of postnatal depression, to collect and collate the specific recommendations reported and examine their quality. |

| 1. **a) Eligibility Criteria for including studies in the review**   This review will focus on published clinical practice guidelines (CPG) for the management of postnatal depression in clinical practice. The inclusion criteria will be (1) Clinical Practice Guidelines published by a recognized national or international association or professionals and developed and endorsed by an expert group. (2) published in the English language; (3) in full-text format; (4) published from 2012 until October 2023 due to advancement in perinatal research and clinical practice guidelines over the last ten years. | |
| --- | --- |
| - - 1. **Population, or participants and conditions of interest** | Women with or at risk of postnatal depression |
| - - 1. **Interventions/Exposure/item of interest** | Any Intervention both pharmacological and non-pharmacological that is aimed at treating postnatal depression recommended in the CPGs. |
| - - 1. **Comparisons or control groups if any** | NA |
| - - 1. **Outcomes of interest** | Main outcome (s)  The recommendations provided in CPG for the improvement of mental health during the postnatal period, pharmacological and non-pharmacological interventions for treatment of PND.  Additional Outcome(s):  The quality of evidence underlying the recommendations |
| - - 1. **Setting** | Community, primary care, and/or specialist inpatient care. |
| - - 1. **Domain or Condition being studied** | Postnatal Depression as defined by standard diagnostic criteria, International Classification Disease (ICD-10), as the onset of depressive symptoms within six weeks of childbirth (Yadav et al., 2020). |

.

| **3. b) Criteria for excluding studies not covered in inclusion criteria**  Any specific populations excluded, date range, language, whether abstracts or full text available, etc |
| --- |
| The exclusion criteria will be: (1) Fact sheets and guidelines intended for patients and other end users but not for healthcare professionals, as these lacked rigor of development or information on stakeholder involvement; (2) guidelines that were not the most recent version; interpretations and reviews of guidelines; (3) Guidelines on conditions such as postnatal psychosis or postnatal anxiety disorders. |

| **4. Search methods** | |
| --- | --- |
| Electronic databases & and websites Please list all databases that are to be searched and include the interface (e.g. NHS HDAS, EBSCO, OVID etc) and date ranges searched for each. | To identify the currently available clinical practice guidelines for the management of PND, a search of both published and grey literature will be done from 2012 to 2023. Initially, a limited search of MEDLINE and relevant published reviews on this subject will be done. This will help to identify and refine index key terms. Based on these initial searches we will identify the clinical practice guidelines for PND.  The following scientific databases will be searched.     - Medline (EBSCO) - PsycINFO (EBSCO) - CINAHL (EBSCO) - TRIP Database - Epistemonikos   Specific websites of the clinical practice guidelines including Guidelines International Network (GIN), World Health Organization (WHO), Scottish Intercollegiate Guidelines Network (SIGN), and National Institute for Health and Clinical Excellence (NICE).  Google will also be included in search.  Search Strategy:  The search strategy will be developed in the Medline database. First, the research question will be broken into two concepts. (1) Clinical Practice Guidelines (2) postnatal depression.  Then keywords and Medical Subject Headings (MeSH) will be identified from the concepts. Inside each concept, keywords and MeSH will be combined by using the Boolean operator OR. Then the two concepts will be combined by using the Boolean operator AND to develop a complete string.  The key search terms will be Clinical Guideline(s) OR guideline(s) OR practice guideline(s) AND Postnatal depression OR postpartum depression. |

| **5. Methods of review** | |
| --- | --- |
| How will search results be managed & documented? ie which reference management software, how duplicates dealt with | Results from each database will be imported into EndNote and duplicates will be removed using Endnote's “remove duplicate” function.  The library will then be transferred to Rayyan to manage the screening process. |
| Selection process Number of reviewers, how agreements to be reached and disagreements dealt with, etc. | In the first stage, two reviewers will independently screen titles and abstracts of retrieved CPGs against the inclusion criteria. Records that do not match the eligibility criteria will be excluded. In the second stage full texts of the selected CPGs will be retrieved and screened by two independent reviewers for inclusion.  Cases of between-reviewer disagreement will be resolved through discussion or where necessary referral to a third reviewer. |
| Quality assessment Tools or checklists used with references or URLs, was this piloted? Is it to be carried out at same time as data extraction? | The Appraisal of Guidelines, Research, and Evaluation version II (AGREE II) will be used to evaluate the quality of the clinical practice guidelines. It consists of 23 items, over six domains which are graded on a 7-point Likert scale. The domains are (i) Scope and purpose of the guideline; (ii) Stakeholder involvement in the development of the guidelines; (iii) Rigour of development and formulation of the recommendations within the guideline; (iv) Clarity of presentation of the guideline; (v) Applicability of the guideline; (vi) editorial independence in the formulation of recommendations within the guideline. AGREE II is an accepted and validated tool for assessing the methodological quality of Guidelines.  Two authors will independently appraise included clinical practice guidelines using the AGREE II instrument. When statements are either unclear or ambiguous, a third author will give the guidance to reach a consensus. Based on the methodological quality of each Guideline, each reviewing author will advocate for or against the use of the Guideline by “recommending the guideline without modifications,” “recommending with modifications,” or “not recommending” the specific guideline”.  As the AGREE II has no classification cut-off, the cut-off of 50% will be applied to indicate those Guidelines statements fulfilling at least 50% of the distinct characteristics of the AGREE II domains. |
| **How is data to be extracted?**  What information is to be collected on each included study? If databases or forms on Word or Excel are used, were these piloted and how is this recorded and by how many reviewers? | Two reviewers will copy all the recommendations verbatim of the included Guidelines and data will be extracted through a data extraction sheet using MS Excel. |
| **Outcomes to be extracted & hierarchy/priority of measures**  ie which measure is preferred and if that is not available which is next in order of preference? | Based on the research questions, the following characteristics will be extracted from them; (1)General characteristics of the guidelines; Title/ author or Institution/Country/ Publication date/ End of search date/Target users/method of reviewing evidence (2) pharmacological interventions: recommended medication/dose/duration/method of administration/adverse events (e.g., relapse in mental illness, toxicity, side effects)/advice on use of concomitant medication (3) non-pharmacological treatment; type, components, follow up period. |
| Narrative synthesis Details of what methods, how synthesis will be done and by whom. Is the Narrative Synthesis Framework to be used? | The results from different clinical practice Guidelines will be summarized to present the consensus statements and recommendations considering the results of the quality appraisal tool and recency of publication.  Data will be narratively described using tables and text. |
| **Meta-analysis**  Details of what and how analysis and testing will be done. If no meta-analysis is to be conducted, please give reason. | As there is no involvement of independent studies so meta-analysis is not applicable here. |
| Will the overall strength of evidence be assessed? If so, how? ie GRADE? | The overall quality of evidence will be assessed using Appraisal of Guidelines, Research, and Evaluation version II (AGREE II). |

| **6. Presentation of results** | |
| --- | --- |
| Outputs from review Papers and target journals, conference presentations, reports, etc | The study's findings will be presented on a variety of platforms, including conferences held locally, nationally, and internationally, as well as peer-reviewed journals. |

**References:**

Abdoli Najmi, L., Mohammad-Alizadeh-Charandabi, S., Jahanfar, S., Abbasalizadeh, F., Salehi Poormehr, H. & Mirghafourvand, M. 2023.Adaptation and implementation of clinical guidelines on maternal and newborn postnatal care in Iran: study protocol.*Reprod Health,*20(1)**,** 135.

Ahmadpour, P., Faroughi, F. & Mirghafourvand, M. 2023.The relationship of childbirth experience with postpartum depression and anxiety: a cross-sectional study.*BMC Psychol,*11(1)**,** 58.

Cheng, Z., Karra, M., Guo, M., Patel, V. & Canning, D. 2023.Exploring the Relationship between Anemia and Postpartum Depression: Evidence from Malawi.*Int J Environ Res Public Health,*20(4).

Dadi, A. F., Miller, E. R. & Mwanri, L. 2020.Postnatal depression and its association with adverse infant health outcomes in low- and middle-income countries: a systematic review and meta-analysis.*BMC Pregnancy Childbirth,*20(1)**,** 416.

Hobeika, E., Malaeb, D., Obeid, S., Salameh, P., Hobeika, E., Outayek, M., Akel, M., Kheir, N., Sleiman, Z., Barakat, H. & Hallit, S. 2023.Postpartum Depression and Anxiety among Lebanese Women: Correlates and Scales Psychometric Properties.*Healthcare (Basel),*11(2).

Hui, C. L. M., Lam, B. S. T., Lee, E. H. M., Chan, S. K. W., Chang, W. C., Suen, Y. N. & Chen, E. Y. H. 2019.A systematic review of clinical guidelines on choice, dose, and duration of antipsychotics treatment in first- and multi-episode schizophrenia.*Int Rev Psychiatry,*31(5-6)**,** 441-459.

Johnston, A., Kelly, S. E., Hsieh, S. C., Skidmore, B. & Wells, G. A. 2019.Systematic reviews of clinical practice guidelines: a methodological guide.*J Clin Epidemiol,*108(64-76.

Keating, D., McWilliams, S., Schneider, I., Hynes, C., Cousins, G., Strawbridge, J. & Clarke, M. 2017.Pharmacological guidelines for schizophrenia: a systematic review and comparison of recommendations for the first episode.*BMJ Open,*7(1)**,** e013881.

Olayemi, E., Asare, E. V. & Benneh-Akwasi Kuma, A. A. 2017.Guidelines in lower-middle income countries.*Br J Haematol,*177(6)**,** 846-854.

Place, J. M., Billings, D. L., Frongillo, E. A., Blake, C. E., Mann, J. R. & deCastro, F. 2016.Policy for Promotion of Women's Mental Health: Insight from Analysis of Policy on Postnatal Depression in Mexico.*Adm Policy Ment Health,*43(2)**,** 189-98.

Wang, Z., Norris, S. L. & Bero, L. 2018.The advantages and limitations of guideline adaptation frameworks.*Implement Sci,*13(1)**,** 72.

Yadav, T., Shams, R., Khan, A. F., Azam, H., Anwar, M., Anwar, T., Siddiqui, C., Abbas, K., Sukaina, M., 2nd & Ghazanfar, S. 2020.Postpartum Depression: Prevalence and Associated Risk Factors Among Women in Sindh, Pakistan.*Cureus,*12(12)**,** e12216.
