# Supplementary material for: Management of postnatal depression: A systematic review of clinical practice guidelines
Source: Glob Ment Health (Camb). 2025 Oct 16;12:e122. doi: 10.1017/gmh.2025.10075 (PMC12641302; doi:10.1017/gmh.2025.10075)
Supplement: Durrani et al. supplementary material [file S2054425125100757sup001.zip › Data_Extraction_sheet_AD_10.10.2024.docx]

| **General Characteristics of the Clinical Practice Guidelines** | | | | | | | |
| --- | --- | --- | --- | --- | --- | --- | --- |
| **SNO.** | **Name of CPG** | **CPG Organization/Society/Institution and Country** | **Publication Date** | **Target Users** | **Guideline review process** | **Level of evidence**  **determination** | **Strength of recommendation** |
| **1** | Treatment and Management of Mental Health Conditions  During Pregnancy and Postpartum | American College of Obstetricians and Gynecologists (ACOG) Clinical Practice Guideline | Jun 2023 | Clinicians in providing obstetric and gynecologic care | Guideline Development Group | Systematic review | GRADE |
| **2** | Antenatal and postnatal mental health: clinical management and service guidance | National Institute for Health & Excellence (NICE) UK | Dec 2014 | Healthcare professionals, Commissioners, Social services, Voluntary and private sectors | Guideline Development Group | Systematic reviews | GRADE |
| **3** | Mental Health Care in the Perinatal Period: Australian Clinical Practice Guideline | Centre of Perinatal Excellence (COPE), Australia | 2023 | all health professionals caring for women and families during the perinatal period. | Guideline Development Group | Systematic review | GRADE |
| **4** | Best Practice Guidelines for Mental Health Disorders in the perinatal Period | Reproductive mental health program, Mental Health and substance use services, and perinatal Services in British Columbia, Canada | Mar 2014 | Healthcare clinicians who care for women during the reproductive years. | Expert Collaboration | Systematic Review | NS |
| **5** | SIGN169: Perinatal Mental health conditions: A national clinical guideline | Healthcare Improvement Scotland (HIS) SIGN | Dec 2023 | family nurse practitioners, general practitioners (GPs), health visitors, midwives, nursery  nurses, nurses, obstetricians, occupational therapists, pharmacists, psychiatrists, psychologists,  psychotherapists, social workers | Guideline development group | Systematic review | GRADE |
| **6** | Perinatal and Infant Mental Health Model of Care – a framework | Government of Western Australia, Dept of Health | 2016 | GP, obstetrician, psychiatrist, neonatologist, midwife, psychologist and  any other involved professionals collaborating actively in developing a perinatal mental health  plan. | Expert Collaboration | NS | NS |
| **7** | British Association for Psychopharmacology consensus guidance on the use of psychotropic  medication preconception, in pregnancy and postpartum | British Association for Psychopharmacology | 2017 | Evidence-based practice. | Guideline Development Group | NS | NS |
| **8** | Perinatal mental health guidelines on depression & anxiety | College of Obstetricians &  Gynaecologists, Singapore | Feb 2023 | GPs, Fam Med/ PHPs, OGYN, Paeds, Nursing, mental health professionals | Expert Collaboration | Systematic Review | NX |
| **9** | Perinatal Depression Screening, Diagnosis, and Treatment guidelines | Kaiser Foundation Health Plan of Washington | April 2021 | NS | Guideline Development Group | Systematic Review | NS |
| **10** | Mental Health Care in the Perinatal Period | Centre of Perinatal Excellence (COPE), Australia | Oct 2017 | The Guideline is intended for all health professionals caring for women and families during the perinatal period. This includes but is  not limited to midwives, general practitioners (GPs), obstetricians, neonatologists, paediatricians, maternal and child health nurses,2  paediatric nurses, Aboriginal and Torres Strait Islander health workers, allied health professionals, mental health practitioners  (psychologists, psychiatrists, mental health nurses, perinatal and infant mental health professionals), consumers and carers and those  working with families in the community (e.g. social workers, child protection agencies), hospital and legal systems. | Expert collaboration | Systematic Review | GRADE |
| **11** | Perinatal Mental Health and Psychosocial Assessment: Practice Resource Manual for Victorian Maternal and Child Health Nurses | Department of Education and Early Childhood Development by the Parent Infant Research Institute (PIRI), Austin Health and Mercy Women’s Hospital Perinatal Mental Health Service, state government Victoria Australia | 2013 | maternal and child health nurses | Guideline Development Group | Systematic literature | NS |
| **12** | Assessment and Interventions for Perinatal Depression | Registered Nurse Association Ontario | Oct 2018 | nurses, the interprofessional team (including, but not limited to physicians, midwives, social workers, lactation consultants, and psychologists), educators, policy-makers, and persons and their families to improve outcomes. | Expert collaboration | Systematic review | AGREE II |
| **13** | The Perinatal Mental Health Care Pathways. Full implementation guidance. | National Collaborating Centre for Mental Health. London | 2018 | clinical  commissioning group (CCG) mental health  commissioners and providers of perinatal  mental health services, statutory and nonstatutory social care providers and local  authorities, working collaboratively with  women who use perinatal mental health  services and their families and carers | Expert collaboration | NS | NS |
| **14** | Evidence-Based Clinical Practice Guidelines for Prevention, Screening and Treatment of Peripartum Depression | This publication is based upon work from the COST Action Research Innovation and Sustainable Pan-European Network in Peripartum Depression Disorder (Riseup-PPD), CA18138, supported by COST (European Cooperation in Science and Technology). | 2019 | mental  health professionals (MHP) (such as psychiatrists, psychologists, counsellors, psychosomatic  medicine practitioners, and other MHPs), other  healthcare professionals including midwives,  obstetricians/gynaecologists, paediatricians,  nurses, general practitioners, social workers,  pharmacists, and others who play key roles in  developing and implementing interventions  for the prevention, screening, or treatment of  PPD. Further targets for these guidelines also  include politicians, economists, policy makers,  and non-profit organisations, including patient organisations, who may be involved  in decision-making about funding, deve–  loping and implementing interventions for  preventing, screening, or treating PPD | Guideline development Group | Systematic Review | GRADE |
| **15** | Perinatal Mental Health and Psychosocial Assessment: Practice Resource Manual for Victorian Maternal and Child Health Nurses | Department of Education and Early Childhood Development by the Parent Infant Research Institute (PIRI), Austin Health and Mercy Women’s Hospital Perinatal Mental Health Service, state government Victoria Australia | 2019 | maternal and child health nurses | Guideline Development Group | Systematic literature | NS |
| **16** | Detection and management of mood disorders in the maternity setting: The Australian Clinical Practice Guidelines | Perinatal and Women’s Mental Health Unit, St John of God Hospital, Australia | 2013 | range of clinicians — midwives, early childhood nurses,  general practitioners, obstetricians, mental health professionals — caring for women and their families across the  perinatal period and cover the detection and management of  perinatal mood disorders, anxiety and psychosis | Expert Collaboration | Systematic review | GRADE |
| **17** | SIGN 127 • Management of perinatal mood disorder | SIGH | March 2012 | midwives, health visitors, general practitioners, pharmacists, psychiatric  nurses, psychiatrists, obstetricians, neonatologists, pediatricians, clinical psychologists, social workers,  public health physicians, users of services, and all other professionals caring for women and their families.  It will also be of value to those commissioning services | Guideline Development Group | Systematic review | GRADE |
| **18** | Maternal Mental Health Guidelines for Healthcare Providers | National Mental Health Programme (NMHP) at the Ministry of Public Health (MOPH), in collaboration with the United  Nations Population Fund (UNFPA) in Lebanon, | OCT 2021 | Obstetric gynecologists • Midwives • Nurses • Pediatricians | Expert Collaboration | Systematic Review | GRADE |
| **19** | Guide for integration of  perinatal mental health in  maternal and child health services | WHO | 2022 | They include clinical managers and health service administrators.  at hospitals, district and primary health facilities and in nongovernmental  organizations (NGOs) and community-based organizations that provide  MCH services. It will also be a useful resource for health-care providers and  allied health professionals. | Guideline Development Group | NS | NS |

| **Pharmacological Interventions** | | | | | | |
| --- | --- | --- | --- | --- | --- | --- |
| **S.No** | **Recommended Medication** | **Dose** | **Duration** | **Method of Administration** | **Adverse Events e.g., Relapse in mental illness, toxicity, side Effects** | **Advice** |
| **1** | First line Rx: SSRIs: Sertraline, Fluoxetine, Citalopram, escitalopram Second line Rx: SNRIs: Duloxetine, venlafaxine, Fluvoxamine, Paroxetine, mirtazapine, bupropion HCL,  A neurosteroid antidepressant: Brexanonlone | SSRIs: Sertraline: 25mg qAM (if sedating change to qHS) Initial increase after 4 days increase to 50mg, Second increase after 7 more days increase to 100mg, Reasses Monthly (increase as needed until symptoms remit) Increase by 50mg, Therapeutic Range*** 50-200mg Fluxetine: 10mg qAM, Initial increase after 4 days increase to 20mg, Reasses Monthly (increase as needed until symptoms remit) Increase by 20mg, Therapeutic Range*** 20-80mg Citalopram: 10mg qAM Initial increase after 4 days increase to 20mg, Reasses Monthly (increase as needed until symptoms remit) Increase by 10mg, Therapeutic Range*** 20-40mg Escitalpram: 5mg qAM, Initial increase after 4 days increase to 10mg, Reasses Monthly (increase as needed until symptoms remit) Increase by 10mg, Therapeutic Range*** 10-20mg Individualized approach to titration: Slower Titration (e.g., every 10-14 days ) is often needed for patients who are antidepressant naive. SNRIs: duloxetine: 30mg*** qAM Initial increase after 4 days increase to 60mg, Reasses Monthly (increase as needed until symptoms remit) Increase by 30mg, Therapeutic Range*** 30-120mg, Venalafaxine: 37.5 mg qAM, Initial increase after 4 days increase to 75mg, Reasses Monthly (increase as needed until symptoms remit) Increase by 75mg, Therapeutic Range*** 75-300mg, Fluvoxamine: 25mg qAM, Initial increase after 4 days increase to 50mg, Second increase after 7 more days increase to 100mg Reasses Monthly (increase as needed until symptoms remit) Increase by 50mg, Therapeutic Range*** 50-200mg, Paroxetine: 10mg***qAM (if sedating change to qHS), Initial increase after 4 days increase to 20mg, Reasses Monthly (increase as needed until symptoms remit) Increase by 10mg, Therapeutic Range*** 20-60mg, mirtazaoine: 7. 5mg qHS, Initial increase after 4 days increase to 15mg, Reasses Monthly (increase as needed until symptoms remit) Increase by 15mg, Therapeutic Range*** 15-45mg, bupropion HCL: 150mg qAM, Reasses Monthly (increase as needed until symptoms remit) Increase by 150mg, Therapeutic Range*** 300-450mg Individualized approach to titration: Slower Titration (e.g., every 10-14 days ) is often needed for patients who are antidepressant naive. For Brexanolone: requires an IV infusion over 60 hours. | NS | Oral | Temporary (days to weeks): Nausea, constipation/Diarrhea lightheadedness, headaches Long-term 9weeks to months): Increased appetite/weight gain, sexual side effects, vivid dreams/insomnia, **QTC prolongation (citalopram & escitalopram) | Tell women to take medication (SSRIs and SNRIs) with food and only increase the dose if tolerating; otherwise, wait until side effects dissipate before increasing. Start medication in the morning, if the patient finds it sedating recommend that she takes it at bedtime. Use of Brexanolone: If the onset of depression occurs in 3rd trimester through 4 weeks postpartum and if the patient is less than 6 months postpartum at screening, consider brexanolone (IV infusion) over 60 hours in an inpatient setting. |
| **2** | TCAs, SSRIs, (S)NRIs | NS | NS | Oral | The risk of discontinuation symptoms in the woman and neonatal adaptation syndrome in the baby with most TCAs, SSRIs and (S)NRIs, in particular paroxetine and venlafaxine. | When psychotropic medication is started in pregnancy and the postnatal period, consider seeking advice, preferably from a specialist in perinatal mental health, and:  • choose the drug with the lowest risk profile for the woman, fetus and baby, taking into account a woman's previous response to medication  • use the lowest effective dose (this is particularly important when the risks of  adverse effects to the woman, fetus and baby may be dose related), but note that sub-therapeutic doses may also expose the fetus to risks and not treat the mental health problem effectively  • use a single drug, if possible, in preference to 2 or more drugs. |
| **3** | SSRIs | NS | NS | Oral | Before choosing a particular antidepressant for pregnant women, consider the woman’s past response to antidepressant treatment,  obstetric history (e.g. other risk factors for miscarriage, preterm birth or postpartum haemorrhage) and any factors that may increase.  risk of adverse effects. | Before prescribing antidepressants to women who are breastfeeding, consider the infant’s health and gestational age at birth. |
| **4** | SSRIs, SNRIs, TCAs | SSRIs:Citalopram 10-40mg per day, Escitalopram 5-20 mg per day, Fluoxetine 20-80mg per day, Fluvoxamine 50-300mg per day, Paroxetine 20-60 mg per day, Sertraline 25-200mg per day, SNRIs: Duloetine 30-120 mg per day, Desvenlafaxine 50-100mg per day, Venlafaxine 37.5-225mg per day, TCAs: Amitriptyline 75-200mg per day, Clomipramine 25-200mg per day, Nortriptyline 75-150mg per day, Others Bupropion 150-300mg per day | NS | Oral | NS | For pregnant women/birthing parents with severe depression, who are taking a TCA, SSRI or SNRI, consider: • continuing their current treatment • changing medication if there is a drug that is effective with a lower risk of adverse effects • combining medication with high intensity psychological intervention such as CBT, or • switching to high intensity psychological intervention if they decide to stop the medication. |
| **5** | SSRIs, SNRIs, TCAa | NS | NS | Oral | NS | NS |
| **6** | NS | NS | NS | NS | NS | NS |
| **7** | NS | NS | NS | NS | NS | General principles of prescribing are outlined in evidence-based guidelines such as the Maudsley Prescribing Guidelines155 and the NICE guidelines69 and include:  Use the lowest effective dose. Avoid using multiple medications wherever possible. Monitor the infant for any specific known adverse effects of the drug. If possible, time the feeds to avoid peak drug levels in breast milk. Note that preterm infants and infants with renal, hepatic, cardiac or neurological impairment are at higher risk from drug exposure. Keep up-to-date with recent developments in the breastfeeding literature. Consider the relative benefits of breastfeeding or formula feeding for each mother and infant, in consultation with the mother and partner |
| **8** | SSRIs | NS | NS | NS | NS | There is substantial evidence that women who are depressed in pregnancy are at an increased risk of depression postnatally. For these women, if antidepressant treatment is required in pregnancy, it is likely to be of benefit for it to continue in the postpartum period.  • Women who are well in pregnancy, but who have had a previous episode of depression postnatally, may also be at  increased risk of subsequent postnatal recurrence. However, there is limited evidence for pharmacological  interventions that can reduce this risk and decisions should be made on an individual basis.  • Pharmacological treatment of depression postnatally does not differ substantially from depression at other times. The most distinctive consideration is in relation to  breastfeeding (see Recommended principles of prescribing to women in the perinatal period: Postnatally and  during breast feeding).  \| As in choosing medication in pregnancy, there is a different risk/benefit analysis to be made depending on whether a woman is initiating antidepressant treatment or is already taking medication. For  initiation, sertraline is associated with low transfer into breastfed babies and associated adverse effects.  However, the risks of other antidepressants may be outweighed by their clinical advantages for continuation treatment.  • It makes good clinical sense that choice of antidepressant in pregnancy takes into account breastfeeding intentions, avoiding the need to consider alteration of  treatment in the early postpartum period, at a time of heightened risk. |
| **9** | Antidepressants | NS | NS | NS | NS | Medication use in postnatal depression/ anxiety  i) Provide counselling on the risk and benefits of starting pharmacological treatment, including potential consequences of untreated depression/ anxiety and adverse side  effects of antidepressants.  ii) Provide support for women in their decision about breastfeeding and be aware that antidepressant use is not an absolute contraindication to breastfeeding. |
| **10** | SSRIs (Sertraline, Escitalopram, Citalopram, paroxetine, fluoxetine, venlafaxine, bupropion | 1st line drug: Sertraline: Initial dose: 50 mg daily x 7 days, then increase to  100 mg daily, Titration schedule  If an unsatisfactory clinical response  after 2–4 weeks, then: Increase by 50 mg increments at  4-week intervals. The usual therapeutic dose range 50-200mg, | NS | NS | NS | NS |
| **11** | SSRIs | NS | NS | NS | NS | Moderate to severe Depression: Use SSRIs as first-line treatment for moderate to severe depression in postnatal women. Before prescribing SSRIs to women who are breastfeeding, consider the infant’s health and gestational age at birth. |
| **12** | NS | NS | NS | NS | NS | Severe symptoms • Mental Health assessment is recommended • Psychological therapies including Cognitive Behavioral Therapy, Interpersonal Psychotherapy, Psychodynamic therapy are recommended and possibly mother infant psychotherapy • Pharmacological treatment. Consider potential risks and benefits to the woman and infant of treatment V’s non-treatment |
| **13** | NS | NS | NS | NS | NS | Pharmacological Nursing Considerations for Perinatal Depression  In collaboration with the interprofessional team, nurses must be cognizant of the use of pharmacological approaches  for perinatal depression, which include the following:   Pharmacological approaches are generally for those with moderate to severe depression or for those whose  depression has not responded to psychological or other non-pharmacological approaches.   Episodes of depression can vary and may have spontaneous remissions, but they can still leave a person at risk for  recurrence.   Safety concerns about the presence or absence of pharmacological treatment may vary across the perinatal period,  and by the individual person.   Discussions pertaining to pharmacological approaches may differ depending on whether the person is pregnant or  lactating.   Each person must be made aware of potential side-effects of medications, their interactions with other  medications, and delays in the onset of a response. Until a response from medications becomes effective, additional  support is recommended.   Medications should be chosen with the lowest known risk.   Dosages of medications for perinatal depression may need to be adjusted due to the pharmacokinetics and  pharmacodynamics of pregnancy and lactation.   Discontinuation of medications can increase the risk of worsening symptoms and relapse of illness.   The understanding of long-term pediatric neurodevelopmental effects is very limited and more evidence in this  area is needed.  The presence or absence of pharmacological, versus non-pharmacological, treatments for perinatal depression may  impact the interactions and bonding between a person and their infant |
| **14** | It is strongly recommended to treat depression during the postpartum period with antidepressant medication, after careful consideration of individual risk-benefit ratio for each woman and her child if breastfed. The decision-making about antidepressant intervention should consider the history of depression recurrence and severity in the woman, previous response to the intervention, and individual preference as well as the health condition of the breastfed child. |  |  |  |  | It is strongly recommended to use brexanolone for moderate to severe postpartum depression treatment if available and if accepted as a treatment option by the woman. |
| **15** | NS | NS | NS | NS | NS | NS |
| **16** | Pharmacological considerations:   1. General   Given paucity of evidence base in perinatal population, consult general population psychotropic medication guidelines for specific dosages, efficacy, etc. Antenatal use: undertake general discussion of potential risks and benefits to woman and foetus of treating versus not treating Antenatal use and birth defects: provide woman and partner detailed explanation of baseline, absolute and relative risks of using specific medication Postnatal use: weigh medication use against minimal possible exposure to infant through breastfeeding If a collaborative decision is made to cease psychotropic medication: slowly taper, monitor closely and plan for early relapse identification or withdrawal symptoms   1. SSRIs   Use of SSRI can be considered in pregnancy as this is the best-researched antidepressant category, and current evidence shows no consistent pattern of additional risk for birth defects (above baseline 2% seen in the general population). Breastfeeding while using SSRIs is not contraindicated in healthy, full-term infants. (c) Mood stabilisers  Valproate: should not be prescribed for bipolar women of childbearing age. Exposure in pregnancy is associated with increased risk of major birth defects and adverse cognitive outcomes for the infant.  Lithium: use with particular caution in breastfeeding due to variable passage into breast milk. Where possible make decision with specialist and organise for ongoing infant monitoring.  (d) Benzodiazepines Can be considered as a short-term option for antenatal and postnatal use, while waiting for the onset of action of an SSRI or TCA in treating anxiety disorders. Avoid long-acting benzodiazepines as much as possible. | NS | NS | NS | NS | NS |
| **17** | Selective serotonin reuptake inhibitors and tricyclic antidepressants may be offered for the treatment of moderate to severe postnatal depression, but with additional considerations regarding the use of antidepressants when breast feeding. Avoid doxepin for treatment of depression in women who are breast feeding. If initiating selective serotonin reuptake inhibitor treatment in breast feeding, then fluoxetine, citalopram and escitalopram should be avoided if possible. | NS | NS | NS | NS | NS |
| **18** | SSRIs are used as first-line treatment for moderate to severe depression postnatally. The risks associated with severe depression on mother-infant bonding outweigh the minimal risk of exposure for the infant through breastmilk. Sertraline and Paroxetine are considered safe while Fluoxetine is not recommended during breastfeeding. |  |  |  |  |  |
| **19** | Fluoxetine  From WHO Essential medicine list 2023 | 20mg as Hydrochloride | NS | Oral | NS | Most women who experience mental health conditions can be helped with The minimum psychotropic medicines that WHO suggests for a health system are listed in the WHO Model List of Essential Medicines. Prescription of psychotropic medicine may, however, be necessary for women with moderate-to-severe symptoms of mental illness, women who are not helped by psychological interventions (or if these are not available) and women with a diagnosis of bipolar disorder, schizophrenia or another form of psychosis. |

| **Non-pharmacological Interventions** | | | | | | |
| --- | --- | --- | --- | --- | --- | --- |
| **S.No.** | **Type** | **Components** | **Duration** | **Follow up Period** | **Monitoring plan** | **General Advice** |
| 1 | CBT or interpersonal therapy | NS | NS | NS | NS | NS |
| 2 | CBT | NS | NS | NS | NS | NS |
| 3 | cognitive behavioral therapy or interpersonal psychotherapy, Directive counselling | NS | NS | NS | NS | NS |
| 4 | A. Psychoeducation,B. Self-Care: The NEST-S Program C. Psychotherapies: CBT, IPT, Psychodynamic therapy, Group therapy, Parent-infant Psychotherapy, Couples and family therapy D. Bright Light Therapy | NS | NS | NS | NS | NS |
| 5 | cognitive behavioural therapy (CBT) and interpersonal therapy (IPT), online interventions (CBT, including internet- and app-based, self directed or clinician guided), directive counselling (including supportive listening, problem solving and goal setting), other-infant relationship interventions and mindfulness. |  |  |  |  |  |
| 6 | cognitive behaviour therapy (CBT) (this includes trauma-focused CBT), eye movement desensitisation reprocessing (EMDR), interpersonal therapy (IPT), dialectical behaviour therapy (DBT), dynamic interpersonal therapy (DIT), mentalisation-based therapy (MBT), transference focused psychotherapy(TFP), systemic family therapy (FT), behavioural couples therapy (BCT) and parent-infant psychotherapy (PIP). | NS | NS | NS | NS | NS |
| 7 | CBT or interpersonal therapy | NS | NS | NS | NS | NS |
| 8 | CBT | NS | NS | NS | NS | NS |
| 9 | cognitive behavioural therapy or interpersonal psychotherapy, | NS | NS | NS | NS | NS |
| 10 | CBT | NS | NS | NS | NS | NS |
| 11 | cognitive behavioural therapy or interpersonal psychotherapy | NS | NS | NS | NS | Mild to Moderate depression: Provide structured psychoeducation to women with symptoms of depression in the perinatal period. Advise women with symptoms of depression in the postnatal period of the potential benefits of a social support group. Recommend individual structured psychological interventions (cognitive behavioural therapy or interpersonal psychotherapy) to women with mild to moderate depression in the perinatal period. Advise women with symptoms of depression in the perinatal period of the potential benefits of facilitated self-help. Advise women with depression or anxiety disorder in the postnatal period of the possible  benefits of directive counseling. |
| 12 | CBT | NS | NS | NS | NS | For women with mild to moderate symptoms • Consider mental health further assessment • Provide psychosocial support including lifestyle advice and early postnatal care, non-directive counselling and possibly peer support • Psychological therapies including Cognitive Behavioural Therapy, Interpersonal Psychotherapy, Psychodynamic therapy are recommended and possibly mother infant psychotherapy • Pharmacological treatment. Consider potential risks and benefits to the woman and infant of treatment vs non-treatment. |
| 13 | Various psychotherapies—including non-directive counselling, CBT, and Interpersonal Therapy (IPT)—were found  to be effective as prevention strategies. | Six sessions | NS | NS | NS | CONCEPTS DISCUSSED One  Introduction;  Symptoms of depression;  Goal setting; and  The relationship between thoughts, feelings, and behaviours. Two  Stress reduction, coping, and relaxation techniques;  Self-monitoring of distorted and negative thinking; and  Increasing positive self-talk and affirmations. Three  Enhance communication skills in the person’s social network;  Evaluating relationships; and  Intimate partner violence and safety planning. Four  Enhancing open communication in the person’s relationships;  Risk factors for prenatal depression and what can help mitigate these risks; and  Signs and symptoms of postpartum depression. Five  Grief, loss and spirituality; and  Practice negative thought-stopping techniques and increase positive self-talk and affirmations. Six  Signs and symptoms of postpartum depression;  Evaluation of the six sessions; and  Goal setting to manage role transition to parenthood. |
| 14 | Cognitive-behavioural therapy (CBT) is strongly recommended for the treatment of depressive symptoms during pregnancy and postpartum. | NS | NS | NS | NS | Third wave CBT therapies, including behavioural activation and mindfulness techniques, are weaklyrecommended for the  treatment of depressive symptoms during pregnancy and postpartum. Interpersonal therapy (IPT) is weakly recommended for the treatment of depressive symptoms during pregnancy and postpartum. ECT is strongly  recommended for the  treatment of therapy  resistant or life-threatening  severe depression in the  postpartum period. |
| 15 | CBT | NS | NS | NS | NS | NS |
| 16 | Non-directive counselling in the context of home visits can be considered as part of the management of mild to moderate depression for women in the postnatal period. Cognitive behavioural therapy (CBT) should be considered for treating women with diagnosed mild to moderate depression in the postnatal period. Interpersonal therapy (IPT) can be considered for treating women with diagnosed mild to moderate depression in the postnatal period. Psychodynamic therapy can be considered for treating women with diagnosed mild to moderate depression in the postnatal period. | NS | NS | NS | NS | NS |
| 17 | Cognitive behavioural therapies should be considered for treatment of mild to moderate depression in the postnatal period. | NS | NS | NS | NS | NS |
| 18 | The most evidence-based interventions for anxiety and depression are Cognitive Behavioral Therapy (CBT) and Interpersonal Psychotherapy (IPT). Both CBT and IPT have been shown to decrease depression in the perinatal period. Many agencies have developed treatment manuals for the use of CBT and IPT for depression. WHO has three relevant manuals: 1. Problem Management Plus1 , which describes the use of behavioural activation, relaxation training, problem solving treatment and strengthening social supports; 2. Group Interpersonal Therapy (IPT) for Depression manual is for the group treatment of depression2and 3. “Thinking Healthy” which is a CBT for perinatal depression. | NS | NS | NS | NS | NS |
| 19 | Behavioral activation: Psychological treatment to improve mood by re-engaging in activities that are task-oriented and used to be enjoyed, despite the current low mood. It may be used as a stand-alone treatment and is also a component of cognitive behavioral therapy (CBT).  Relaxation training; Training in techniques such as breathing exercises to bring about a relaxation response.  Problem-solving treatment: Psychological treatment that involves systematic problem-identification and problem-solving techniques in several sessions.  Interpersonal therapy: Psychological treatment by linking depressive symptoms and interpersonal problems, especially those involving grief, disputes, life changes and social isolation. Also known as “interpersonal psychotherapy”.  Cognitive behavioral therapy (CBT): Depression, substance use conditions, psychoses, trauma, self-harm, suicide. Psychological treatment comprises cognitive components (for thinking differently, for example, by identifying and challenging unrealistic negative thoughts) and behavioral components (doing things differently, for example, helping a person to do more rewarding activities). CBT for trauma usually includes exposure (in images or in person) and/or a direct challenge to unhelpful trauma-related thoughts and beliefs.  Parenting skills training: A group of treatment programmes to change care-giving behaviour and strengthen confidence in using effective caregiving strategies. It involves teaching caregivers emotional communication, positive caregiver–child interaction skills, and positive reinforcement methods to improve the behaviour and functioning of children and adolescents. |  |  |  |  | Conditions with mild symptoms  Brief psychological interventions for women in the perinatal period with suspected common mental health  conditions such as depression or anxiety can be provided by trained MCH service providers. WHO has published manuals on the use of the following evidence-based interventions:  Thinking Healthy (perinatal depression)  Problem Management Plus (PM+) (depression, anxiety and stress)  Group Interpersonal Therapy (depression)  Self-Help Plus (SP+) (stress)  Conditions with moderate-to-severe symptoms  Severe mental health conditions include psychosis, bipolar disorder, suicidality, and severe depression and are characterized by disordered thinking and behavior. Women with moderate-to-severe mental health conditions have several symptoms of mental ill health that significantly affect their ability to engage in daily activities (including care of their infant), most of the time, on most days, for at least 2 weeks.  These conditions usually require more intensive interventions that are delivered or supervised by mental health specialists. |
