# Supplementary material for: Management of postnatal depression: A systematic review of clinical practice guidelines
Source: Glob Ment Health (Camb). 2025 Oct 16;12:e122. doi: 10.1017/gmh.2025.10075 (PMC12641302; doi:10.1017/gmh.2025.10075)
Supplement: Durrani et al. supplementary material [file S2054425125100757sup001.zip › Search_strategy_05.10.2024_AD.docx]

**Clinical Practice Guidelines for Postnatal Depression: A Systematic Review**

| **S.No.** | **Databases** | **Hit** |
| --- | --- | --- |
| 1 | Medline (EBSCO) | 333 |
| 2 | CINAHIL (EBSCO) | 243 |
| 3 | PsycINFO (EBSCO) | 142 |
| 4 | Epistemonikos | 340 |
| 5 | Trip Database | 20 |
| 6 | Guidelines International Network (G-I-N) | 4 |
| 7 | National Institute for Health and Clinical Excellence (NICE) | 4 |
| 8 | SIGN | 2 |
| 9 | WHO | 1 |
| 10 | Google | 7 |
|  | **Total** | **1096** |

**Search Strategy for Medline (EBSCO run 05.10.2024)**

| S42 | S21 AND S40  **Limiters** - Publication Date: 20120101-20231231 | (333) |
| --- | --- | --- |
| S41 | S21 AND S40 | (482) |
| S40 | S22 OR S23 OR S24 OR S25 OR S26 OR S27 OR S28 OR S29 OR S30 OR S31 OR S32 OR S33 OR S34 OR S35 OR S36 OR S37 OR S38 OR S39 | (520,995) |
| S39 | AB (care n2 (standard or standards)) and (guideline* OR guidance OR recommendation*) | (11,472) |
| S38 | AB (care n2 (path or paths or pathway or pathways or map or maps or plan or plans)) and (guideline* OR guidance OR recommendation* OR standard*) | (8,881) |
| S37 | AB ((clinical or critical or practice) n2 (path or paths or pathway or pathways or protocol*)) and (guideline* OR guidance OR standard* OR recommendation*) | (9,773) |
| S36 | TI practice n2 parameter* OR AB practice n2 parameter* | (1,949) |
| S35 | TI "policy statement*" OR AB "policy statement*" | (2,705) |
| S34 | TI "position statement*" OR AB "position statement*" | (5,445) |
| S33 | (MH "Critical Pathways") | (7,905) |
| S32 | AB treatment* n2 (guideline* OR standard* OR recommendation*) | (107,852) |
| S31 | AB clinical w0 (guideline* OR standard* OR recommendation*) | (21,800) |
| S30 | AB practice w0 (guideline* OR standard* OR recommendation*) | (31,534) |
| S29 | TI (practice or treatment* OR clinical) n2 consensus | (1,666) |
| S28 | TI recommendation* | (51,327) |
| S27 | TI (practice or treatment* OR clinical) w0 standard | (625) |
| S26 | TI standards | (78,826) |
| S25 | TI guidance | (21,867) |
| S24 | TI guideline* | (95,765) |
| S23 | (MH "Practice Guidelines as Topic") | (127,701) |
| S22 | (MH "Guidelines as Topic+") | (172,951) |
| S21 | S19 OR S20 | (22,656) |
| S20 | (MH "Depression, Postpartum") | (7,645) |
| S19 | S14 AND S18 | (22,656) |
| S18 | S15 OR S16 OR S17 | (662,297) |
| S17 | (MH "Depressive Disorder+") | (123,239) |
| S16 | (MH "Depression") | (153,946) |
| S15 | AB depress* OR TI depress* OR SU depress* | (661,521) |
| S14 | S1 OR S2 OR S3 OR S4 OR S5 OR S6 OR S7 OR S8 OR S9 OR S10 OR S11 OR S12 OR S13 | (372,770) |
| S13 | AB intrapartum OR TI intrapartum OR SU intrapartum | (10,798) |
| S12 | AB antepartum OR TI antepartum OR SU antepartum | (6,780) |
| S11 | AB antenatal OR TI antenatal OR SU antenatal | (45,592) |
| S10 | AB perinatal OR TI perinatal OR SU perinatal | (92,099) |
| S9 | AB postnatal OR TI postnatal OR SU postnatal | (125,992) |
| S8 | AB postpartum OR TI postpartum OR SU postpartum | (87,556) |
| S7 | AB ante W0 partum OR TI ante W0 partum OR SU ante W0 partum | (489) |
| S6 | AB Intra W0 partum OR TI Intra W0 partum OR SU Intra W0 partum | (394) |
| S5 | AB ante W0 natal OR TI ante W0 natal OR SU ante W0 natal | (659) |
| S4 | AB peri W0 natal OR TI peri W0 natal OR SU peri W0 natal | (222) |
| S3 | AB Post W0 natal OR TI Post W0 natal OR SU Post W0 natal | (8,548) |
| S2 | AB Post W0 partum OR TI Post W0 partum OR SU Post W0 partum | (13,765) |
| S1 | (MH "Postpartum Period+") | (76,838) |

**Search Strategy for CINAHIL (EBSCO run 05.10.2024)**

| S40 | S20 AND S38  **Limiters** - Publication Date: 20120101-20231231 | (243) |
| --- | --- | --- |
| S39 | S20 AND S38 | (341) |
| S38 | S21 OR S22 OR S23 OR S24 OR S25 OR S26 OR S27 OR S28 OR S29 OR S30 OR S31 OR S32 OR S33 OR S34 OR S35 OR S36 OR S37 | (232,572) |
| S37 | AB (care n2 (standard or standards)) and (guideline* OR guidance OR recommendation*) | (5,051) |
| S36 | AB (care n2 (path or paths or pathway or pathways or map or maps or plan or plans)) and (guideline* OR guidance OR recommendation* OR standard*) | (5,037) |
| S35 | AB ((clinical or critical or practice) n2 (path or paths or pathway or pathways or protocol*)) and (guideline* OR guidance OR standard* OR recommendation*) | (3,555) |
| S34 | TI practice n2 parameter* OR AB practice n2 parameter* | (701) |
| S33 | TI "policy statement*" OR AB "policy statement*" | (1,635) |
| S32 | TI "position statement*" OR AB "position statement*" | (4,212) |
| S31 | (MH "Critical Path") | (6,050) |
| S30 | AB treatment* n2 (guideline* OR standard* OR recommendation*) | (29,957) |
| S29 | AB clinical w0 (guideline* OR standard* OR recommendation*) | (9,200) |
| S28 | AB practice w0 (guideline* OR standard* OR recommendation*) | (16,350) |
| S27 | TI (practice or treatment* OR clinical) n2 consensus | (673) |
| S26 | TI recommendation* | (23,306) |
| S25 | TI (practice or treatment* OR clinical) w0 standard | (487) |
| S24 | TI standards | (35,635) |
| S23 | TI guidance | (11,236) |
| S22 | TI guideline* | (50,578) |
| S21 | (MH "Practice Guidelines") | (86,597) |
| S20 | S18 OR S19 | (13,412) |
| S19 | (MH "Depression, Postpartum") | (7,012) |
| S18 | S14 AND S17 | (13,412) |
| S17 | S15 OR S16 | (230,627) |
| S16 | (MH "Depression+") | (138,240) |
| S15 | AB depress* OR TI depress* OR SU depress* | (230,553) |
| S14 | S1 OR S2 OR S3 OR S4 OR S5 OR S6 OR S7 OR S8 OR S9 OR S10 OR S11 OR S12 OR S13 | (118,159) |
| S13 | AB intrapartum OR TI intrapartum OR SU intrapartum | (6,179) |
| S12 | AB antepartum OR TI antepartum OR SU antepartum | (2,419) |
| S11 | AB antenatal OR TI antenatal OR SU antenatal | (18,637) |
| S10 | AB perinatal OR TI perinatal OR SU perinatal | (45,981) |
| S9 | AB postnatal OR TI postnatal OR SU postnatal | (35,833) |
| S8 | AB postpartum OR TI postpartum OR SU postpartum | (34,440) |
| S7 | AB ante W0 partum OR TI ante W0 partum OR SU ante W0 partum | (46) |
| S6 | AB Intra W0 partum OR TI Intra W0 partum OR SU Intra W0 partum | (103) |
| S5 | AB ante W0 natal OR TI ante W0 natal OR SU ante W0 natal | (185) |
| S4 | AB peri W0 natal OR TI peri W0 natal OR SU peri W0 natal | (49) |
| S3 | AB Post W0 natal OR TI Post W0 natal OR SU Post W0 natal | (1,325) |
| S2 | AB Post W0 partum OR TI Post W0 partum OR SU Post W0 partum | (3,061) |
| S1 | (MH "Postnatal Period+") | (17,676) |

**Search strategy for PsycINFO (EBSCO run 05.10.2024)**

| S43 | S24 AND S41  **Limiters** - Publication Year: 2012-2023 | (142) |
| --- | --- | --- |
| S42 | S24 AND S41 | (235) |
| S41 | S25 OR S26 OR S27 OR S28 OR S29 OR S30 OR S31 OR S32 OR S33 OR S34 OR S35 OR S36 OR S37 OR S38 OR S39 OR S40 | (67,094) |
| S40 | AB (care n2 (standard or standards)) and (guideline* OR guidance OR recommendation*) | (1,435) |
| S39 | AB (care n2 (path or paths or pathway or pathways or map or maps or plan or plans)) and (guideline* OR guidance OR recommendation* OR standard*) | (1,368) |
| S38 | AB ((clinical or critical or practice) n2 (path or paths or pathway or pathways or protocol*)) and (guideline* OR guidance OR standard* OR recommendation*) | (1,015) |
| S37 | TI practice n2 parameter* OR AB practice n2 parameter* | (484) |
| S36 | TI "policy statement*" OR AB "policy statement*" | (708) |
| S35 | TI "position statement*" OR AB "position statement*" | (856) |
| S34 | AB treatment* n2 (guideline* OR standard* OR recommendation*) | (14,146) |
| S33 | AB clinical w0 (guideline* OR standard* OR recommendation*) | (3,982) |
| S32 | AB practice w0 (guideline* OR standard* OR recommendation*) | (6,949) |
| S31 | TI (practice or treatment* OR clinical) n2 consensus | (126) |
| S30 | TI recommendation* | (8,420) |
| S29 | TI (practice or treatment* OR clinical) w0 standard | (67) |
| S28 | TI standards | (11,515) |
| S27 | TI guidance | (7,657) |
| S26 | TI guideline* | (9,043) |
| S25 | MA guideline | (9,712) |
| S24 | S20 OR S23 | (13,543) |
| S23 | S21 OR S22 | (2,782) |
| S22 | MA postpartum depression | (2,782) |
| S21 | MA postnatal depression | (0) |
| S20 | S15 AND S19 | (13,543) |
| S19 | S16 OR S17 OR S18 | (386,948) |
| S18 | MA depressive disorder | (61,525) |
| S17 | MA depression | (58,527) |
| S16 | AB depress* OR TI depress* OR SU depress* | (386,948) |
| S15 | S1 OR S2 OR S3 OR S4 OR S5 OR S6 OR S7 OR S8 OR S9 OR S10 OR S11 OR S12 OR S13 OR S14 | (50,974) |
| S14 | AB intrapartum OR TI intrapartum OR SU intrapartum | (468) |
| S13 | AB antepartum OR TI antepartum OR SU antepartum | (784) |
| S12 | AB antenatal OR TI antenatal OR SU antenatal | (4,531) |
| S11 | AB perinatal OR TI perinatal OR SU perinatal | (14,002) |
| S10 | AB postnatal OR TI postnatal OR SU postnatal | (26,547) |
| S9 | AB postpartum OR TI postpartum OR SU postpartum | (17,503) |
| S8 | AB ante W0 partum OR TI ante W0 partum OR SU ante W0 partum | (13) |
| S7 | AB Intra W0 partum OR TI Intra W0 partum OR SU Intra W0 partum | (15) |
| S6 | AB ante W0 natal OR TI ante W0 natal OR SU ante W0 natal | (68) |
| S5 | AB peri W0 natal OR TI peri W0 natal OR SU peri W0 natal | (78) |
| S4 | AB Post W0 natal OR TI Post W0 natal OR SU Post W0 natal | (1,290) |
| S3 | AB Post W0 partum OR TI Post W0 partum OR SU Post W0 partum | (1,509) |
| S2 | MA postpartum | (4,909) |
| S1 | MA postnatal | (680) |

**Epistemonikos (Run 05.10.2024)**

**Total 340**

title:(perinatal OR postnatal OR Postpartum OR antepartum OR intrapartum) OR abstract:(perinatal OR postnatal OR Postpartum OR antepartum OR intrapartum) AND title:(Depress*) OR abstract:(Depress*) OR title:("Depressive disorder*") OR abstract:("Depressive disorder*")

Use built-in filters

- Publication year: 2012-2023

**Trip Database (Run 05.10.2024).**

Crude search: 660 of which 20 were relevant and downloaded.

Keywords: Postnatal depression OR postpartum depression

Use built-in filters.

- Guidelines
- Publication year: 2012-2023

**Guidelines International Network (G-I-N) (Run 05.10.2024)**

**Total 4**

Keywords: Postpartum depression, Postnatal depression, and Perinatal depression.

**National Institute for Health and Clinical Excellence (NICE) (Run 05.10.2024)**

**Total 4**

Keywords: Postpartum depression, Postnatal depression, and Perinatal depression.

Use built-in filters.

Type: Guidelines

Status: Published

**Scottish Intercollegiate Guidelines (SIGN) (Run 05.10.2024)**

**Total 2**

Keywords: Postpartum depression, Postnatal depression, and Perinatal depression.

**WHO Guidelines (Run 05.10.2024)**

**Total 1**

Key Words: Postpartum depression, Postnatal depression, Perinatal depression

**Google (Run 05.10.2024)**

**Total 7**

The searches using the following keywords were executed in Google.

Guidelines for postpartum Depression=4 (Search on first 10 pages)

Guidelines for perinatal Depression= 2 (Search on first 10 pages)

Guidelines for postnatal Depression= 1 (Search on first 10 pages)

We reviewed the first 10 pages to identify records relevant to our research question.
